# Landmarks for diagnosis and surgery in abnormal ventriculo-arterial connections with usual arrangement

**DOI:** 10.1186/1749-8090-8-S1-O251

**Published:** 2013-09-11

**Authors:** A Capuani, N Soulé, M Meot, B Aupy, MC Vaillant, J Poinsot, B Lefort, A Chantepie, P Marticho, P Neville

**Affiliations:** 1 Ped. Cardiology, Clocheville Hospital, CHRU Tours, France

## Background

The term “malposition of great arteries“ is commonly used for diagnosis and surgery in abnormal ventriculo-arterial connections with usual arrangement and two well developed ventricles. In these abnormalities the variability of the arterial relationships is considerable and all relationships from normal to side by side to antero posterior have to be expected.

Can we find other anatomical landmarks for the diagnosis and the subsequent corrective surgery?

## Methods

We reviewed the specimens previously published and observations collected in three different institutions.

## Results

There is a gradual transition from Tetralogy of Fallot to double outlet right ventricle with subaortic ventricular septal defect to Taussig-Bing hearts to transposition of great arteries. In this transition the normal aortic-mitral continuity is progressively lost with gradual development of pulmonary- mitral continuity.

The key features determining the sequence of these abnormalities are the relationships between the outlet septum and the ventricular musculature. We may describe and group these malformations in function of the anticlockwise rotation of the outlet septum and the septomarginal trabeculation.

## Conclusion

The term “malposition of great arteries“ is a generic term which is of little help for diagnosis and surgery, therefore, it should be avoided.

We recommend a detailed description of the outlet septum and the septomarginal trabeculation in relation to the VSD. Careful attention to these structures dictates the diagnosis and the best surgical approach.

